# Highly Sensitive Strain Sensing Using a Resonant Fiber Bragg Grating

**DOI:** 10.3390/mi17101175

**Published:** 2026-10-10

**Authors:** Eli Ashkenazy, Slava Krylov, Moshe Tur

**Affiliations:** 1 School of Electrical and Computer Engineering, Faculty of Engineering, Tel-Aviv University, Tel-Aviv 6997801, Israel; asheli@live.com (E.A.); tur@tauex.tau.ac.il (M.T.); 2 School of Mechanical Engineering, Faculty of Engineering, Tel-Aviv University, Tel-Aviv 6997801, Israel; 3 Faculty of Engineering, Holon Institute of Technology, Holon 58102, Israel

**Keywords:** strain and force sensing, optical fiber-beam, fiber Bragg grating (FBG), double-clamped beam, resonant sensing, vibrating beam acceleromete

## Abstract

We report on a strain sensing approach that relies on monitoring the mechanical resonant frequency of a suspended, double-clamped, stretched optical fiber with an inscribed Bragg grating (FBG). This resonant frequency, which varies with strain, is measured using the FBG. A reduced-order model of the fiber-beam is used to estimate the expected frequency shift $\Delta f=f-f_{0}$, with respect to an unstretched fiber frequency $f_{0}$, and to determine appropriate design and operating parameters. Using a custom-built setup that provides controlled fiber stretching and excitation by an external shaker, frequency sensing in the kHz range is experimentally demonstrated. The FBG measurements using the slope-assisted interrogation technique are validated by simultaneously detecting the fiber’s time-domain response with a laser Doppler vibrometer (LDV). Consistently with the model prediction, an experimental frequency–strain scale factor of Δf/ε≈5.6Hz/με is obtained. The corresponding normalized sensitivity, $(\Delta f/f_{0})/\varepsilon \approx 682$, is more than two orders of magnitude higher than that obtained by direct FBG strain measurement. This resonance-based approach also retains the potential for multiplexed sensing at multiple locations along a single fiber. The applicability of the technique to resonant vibrating-beam accelerometers is demonstrated using the model.

## 1. Introduction

Resonant devices serve as a core element in a majority of micro- and nanoelectromechanical system (MEMS/NEMS)-based sensors. In these devices, various environmental stimuli affect the mass and/or stiffness properties of a vibrating sensing element and, consequently, its natural frequencies, which are then measured to extract the quantity of interest [1,2,3,4,5]. The resonant sensing principle plays an instrumental role in chemical, bio [6,7,8] mass sensors [9,10], temperature [11] and gas sensors [12], electric and magnetic field sensors [13], pressure sensors [14,15] or condition monitoring devices for industrial machinery predictive maintenance [16,17,18]. Some of these technologies, such as cantilever biosensors [6,7,8], microbalance mass sensors, or atomic force microscopy [19], are well established and have paved their way into commercial products. Others are intensively investigated in order to establish sufficient foundations for the future use of new types of resonant sensors in applications.

Along with significant advances in resonant sensing devices, many remarkable challenges still remain to be addressed. One of these challenges is related to sensing. The operational scenario of resonant sensors requires reliable and highly precise measurement of the vibrating elements’ mechanical response. While several sensing approaches were explored in micro sensors, including piezoresistive [20,21], piezoelectric [4], or magnetic [22,23], electrostatic capacitive sensing remains the most widely implemented [2,24]. However, capacitive sensing involves several challenges, mainly related to the small area of sensing electrodes, the influence of parasitic capacitance, and cross-talk. To suppress the undesired influence of electrostatic nonlinearities and avoid intrinsic instabilities, the resonators are typically operated at small (when compared to the distance between the electrodes) amplitudes, which results in small capacitance variations (often of the order of aF) and makes the detection difficult. Moreover, in structural health monitoring applications [25] or inertial sensor networks for industrial machinery predictive maintenance [16,18], it is often necessary to carry out measurements in multiple locations. This requirement significantly complicates addressing and capacitive interrogation of the individual devices. Optical sensing is considered to be one of the promising alternatives and is intensively investigated [26]. These sensors are often based either on Fabry–Péro interferometry [27,28] or incorporate nanophotonic circuits [29,30]. However, implementation of optical sensing requires the incorporation of light sources (such as lasers or LEDs) and photodetectors and generally complicates device integration, while the realization of nanophotonic devices often imposes pronounced fabrication challenges [26].

One of the successfully implemented approaches, also suitable for reliable sensing in multiple locations, is the fiber Bragg grating technology [31,32,33]. Here, a few millimeters of the fiber core are properly exposed to optical radiation at around 248 nm or to intense pulses from Femtosecond lasers at 800 nm. Either illumination is structured to write a permanent refractive index grating in the fiber core. This periodic structure selectively reflects light at narrow wavelength range (<1 nm) around λ=2nΛ, where *n* and Λ are the local refractive index and grating period, respectively. Both these grating parameters are sensitive to external measurands (such as strain and temperature), resulting in shifts in the wavelength of peak reflection. FBGs have many obvious advantages: (i) they can be multiplexed, i.e., many tens of FBGs can be written on the same fiber at different locations; (ii) modern interrogators can simultaneously read them at high speeds (a few kHz) [32,33], allowing dynamic measurements (up to ultrasonic frequencies in dedicated setups); and (iii) they can be imprinted on the fiber while on the drawing tower, promising low prices. FBGs are nowadays successfully and ubiquitously used in many applications. In particular, FBG-based sensing systems can have nano-strain sensitivity with hundreds of sensing points along tens of kilometers [34,35]. They are used for direct strain or temperature sensing and also for indirect (through conversion of physical stimuli into mechanical strain) sensing of other measurands such as forces, rotations, vibrations, and electrical and magnetic fields.

**Figure 1:**
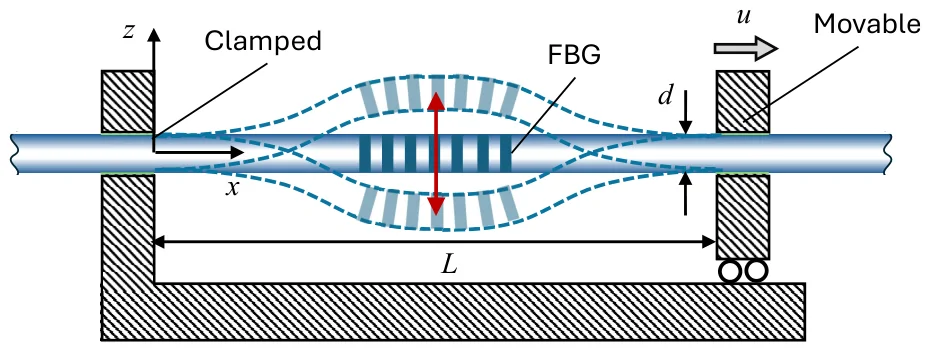
Operational principle of the resonant FBG sensor. The dashed lines depict the fiber in the deformed configuration.

In this work, we propose a novel approach for resonant strain/force sensing, utilizing the FBG fiber itself as the vibrating sensing element. Here, a double-clamped suspended section of a single-mode fiber has an FBG, inscribed within the suspended section, Figure 1. When axial strain is induced in the fiber by a displacement of one of its ends *u*, the fiber’s stiffness is altered, thereby shifting the mechanical resonant frequency, which can then be used for the measurement of strain and other measurands. As is common in resonant sensors, the vibrations of the fiber segment are excited by an actuating force and the strain-dependent resonant frequency shift is detected in the framework of an open-loop (driving frequency sweep) or closed-loop (self-excitation) scenario. Among the key advantages of the proposed approach over capacitive sensing is that it eliminates the need for additional sensing electrodes. It also supports the multiplexed sensing capabilities of optical fibers while retaining the simplicity, robustness, low cost, and high-performance characteristics of FBGs. Most importantly, the proposed approach provides a two-orders-of-magnitude improvement in sensitivity—expressed as the relative frequency shift per unit strain—compared with conventional FBG strain sensors, which rely on the direct strain-induced shift of the FBG peak-reflection wavelength. While the suggested strain sensing approach can be used in a large variety of resonant sensors, in the present work we also explore the feasibility of this FBG-based resonant frequency sensing for an implementation of a resonant accelerometer.

Section 2 presents a detailed mechanical model of the fiber segment, treated as a beam. The experimental setup and the resulting measurements are detailed in Section 3 and Section 4, respectively. Section 5 discusses implementations of the approach as a vibrating beam accelerometer, while Section 6 offers conclusions and directions for further research.

## 2. Model of the Beam

In order to evaluate the feasibility of the strain sensing in the framework of the resonant operation scenario and estimate possible performance, a model of the vibrating beam stretched by an axial force *N* is considered. The silica-made optical fiber is considered as a suspended prismatic double-clamped beam with a circular cross section. The dynamics of such a beam are described in the framework of the Euler–Bernoulli theory combined with a nonlinear Kirchhoff stretching and are governed by [36]

(1) $$EI_{yy}\frac{\partial^{4}w}{\partial x^{4}}+c\frac{\partial w}{\partial t}+\rho A\frac{\partial^{2}w}{\partial t^{2}}-N\frac{\partial^{2}w}{\partial x^{2}}=p_{0}$$

where w(x,t) is the coordinate *x* and time *t*-dependent deflection of the beam in the *z* direction, *E* is the beam’s material Young’s modulus, ρ is the mass density, $A=\pi d^{2}/4$ and $I_{yy}=\pi d^{4}/64$ are the area and the second moment of area of the beam’s cross section, respectively, *c* is the viscous damping coefficient, and $p_{0}\left(t\right)$ is the actuating force assumed to be uniformly distributed along the beam. The axial stretching force

(2) $$N=N_{0}+N_{nl},\;N_{0}=EA\varepsilon_{xx}^{0},\;N_{nl}=EA\varepsilon_{xx}^{nl}=\frac{EA}{2L}\int_{0}^{L}{\left(\frac{\partial w}{\partial x}\right)}^{2}dx$$

contains the constant, deflection-independent component $N_{0}$, and the nonlinear, deflection-dependent component $N_{nl}$ induced by the fiber’s motion. Here $\varepsilon_{xx}^{0}$ is the axial strain to be measured by the sensor and $\varepsilon_{xx}^{nl}$ is the nonlinear, deflection-dependent component of the strain. Both strain components are uniform within the cross section of the fiber. In this work, the material and geometric parameters of the beam are chosen to be consistent with those of a typical commercially available optical fiber and are listed in Table 1. We reiterate that while more advanced models of the device accounting for an additional (fabrication- or assembly-related) pre-stress, imperfections, and the influence of nonlinearities on the dynamic response can be analyzed, our primary goal here is to estimate the beam’s mechanical response and its applicability as a strain sensor.

**Figure 2:**
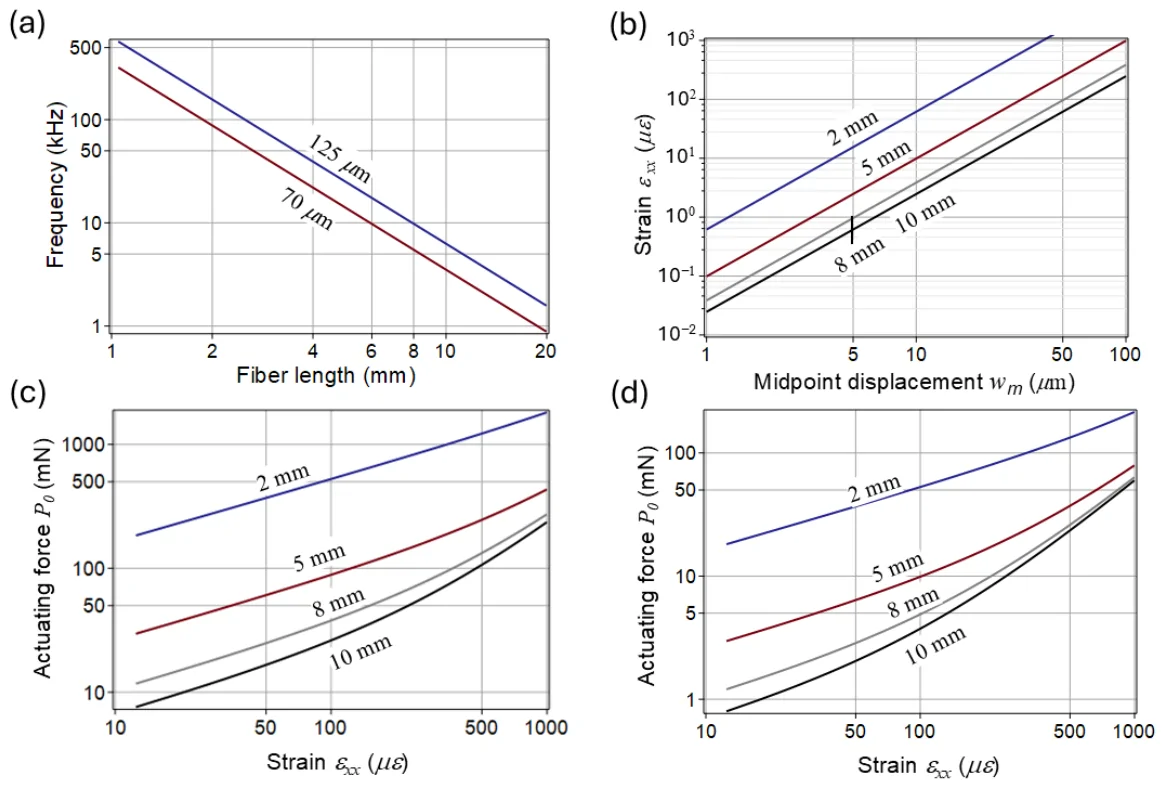
(**a**) Baseline resonance frequency as a function of the suspended fiber length *L* for two diameters *d* of commercially available fibers (numbers). (**b**) Axial strain $\varepsilon_{xx}$ as a function of the fiber midpoint deflection $w_{m}$ for different fiber lengths (numbers). (**c**) Static resultant actuation force $P_{0}$ required to reach different strain levels in the fibers of different lengths (numbers) and of the diameter d=125μm and (**d**) *d* = 70 μm.

In the case of small amplitude vibrations, the fundamental frequency associated with the linear counterpart of Equation (1) (when $N=N_{0}$) can be approximated by the expression [37]

(3) $$f\left(N_{0}\right)\approx f_{0}\;\sqrt{1+\gamma \frac{N_{0}}{N_{E}}}\approx f_{0}\left(1+\frac{\gamma N_{0}}{2N_{E}}\right),\;\left(\frac{N_{0}}{N_{E}}\ll 1\right)$$

where

(4) $$f_{0}=\frac{\lambda_{1}^{2}}{2\pi L^{2}}\sqrt{\frac{EI_{yy}}{\rho A}}=\frac{\lambda_{1}^{2}cd}{8\pi L^{2}}$$

is the natural frequency of the beam without the axial force (N=0), $\lambda_{1}=4.73$ is the fundamental mode eigenvalue, $N_{E}=4\pi^{2}EI/L^{2}$ is the Euler’s buckling force of a double-clamped beam, and $c=\sqrt{E/\rho }=5640$ m/s is the speed of sound in the fiber’s material (SiO_2_). In addition, γ≈0.96 is the correction coefficient, which we take to be unity for simplicity. The baseline (at N=0) fundamental mode frequencies $f_{0}$ as a function of the suspended fiber segment length are shown in Figure 2a for the two diameters of common commercially available fibers.

It is instructive to consider the scale factor SF (sensitivity) of the vibrating fiber in terms of the axial strain $\varepsilon_{xx}^{0}=u/L$ due to the beam’s end displacement *u*. Since the axial force $N_{0}=EA\varepsilon_{xx}^{0}$ is linearly related to the strain, Equation (2), the frequency shift δf, the absolute (SF) and the relative ($SF_{\mathrm{REL}}$) sale factors are

(5) $$\begin{array}{ccc} \Delta f & = & \frac{N_{0}}{2N_{E}}=\frac{c\lambda_{1}^{2}}{4\pi^{3}d}\;\varepsilon_{xx}^{0} \end{array}$$

(6) $$\begin{array}{ccc} SF & = & \frac{\Delta f}{\varepsilon_{xx}^{0}}=\frac{f_{0}EA}{2N_{E}}=\frac{c\lambda_{1}^{2}}{16\pi^{3}}\sqrt{\frac{A}{I_{yy}}}=\frac{c\lambda_{1}^{2}}{4\pi^{3}d} \end{array}$$

(7) $$\begin{array}{ccc} SF_{\mathrm{REL}} & = & \frac{\Delta f}{f_{0}\varepsilon_{xx}^{0}}=\frac{EA}{2N_{E}}=\frac{AL^{2}}{8\pi^{2}I_{yy}}=\frac{2}{\pi^{2}}{\left(\frac{L}{d}\right)}^{2} \end{array}$$

where $\Delta f=\left(f-f_{0}\right)/f_{0}$ is the frequency shift, and $c=\sqrt{E/\rho }=5640$ m/s is the speed of sound in the fiber’s material (SiO_2_). In accordance with Equation (5), in the case of $N/N_{E}\ll 1$, the frequency shift $\Delta f=f-f_{0}$ is linearly proportional to the axial force $N_{0}$ and is independent of the beam’s length *L* [38]. As suggested by Equation (6), the constant (in the first-order approximation: the SF nonlinearity is not addressed in this work) SF is dictated solely by the fiber diameter and the material properties. As a result, for the same silica fiber, the universal length-independent absolute SF values for 125 μm and 70 μm diameter fibers are 8.16 Hz/με and 14.53 Hz/με, respectively. Equation (7), on the other hand, highlights one of the key advantages of the resonant strain sensing approach. Compared to direct strain measurement, for the typical fiber dimensions d/L≈0.02 the relative frequency shift can be orders of magnitude larger than the strain itself. For example, for a standard d=125μm diameter silica fiber of the length L=8 mm, the expected relative scale factor is $SF_{\mathrm{REL}}=830$, implying much better sensitivity than a direct strain measurement.

By considering Equations (4), (6) and (7), geometric and operational parameters of the fiber can be chosen. Optimization of the fiber parameters for performance is out of the scope of the present work since the figures of merit are generally dictated by a specific application. Note, however, that downscaling of the sensor (smaller *d*) leads to higher SF and $SF_{\mathrm{REL}}$. On the other hand, to preserve sufficiently high natural frequency, the fiber length should also be decreased, leading to lower $SF_{\mathrm{REL}}$. Working at higher frequencies allows higher bandwidth and better immunity to noise at lower frequencies as well as smaller flicker noise (1/f). However, measurement of high frequencies using FBG is more challenging and might require using fast interrogation techniques and equipment. In this work, the length of the fiber was chosen within the range between 8 mm and 12 mm, which results in resonant frequencies below 10 kHz.

### Actuation

In order to evaluate the feasibility of resonant strain sensing using FBG, two aspects should be addressed. The first is related to the motion-induced axial strain $\varepsilon_{xx}$, which can be represented as a sum of two components. The bending-related component varies linearly with *z*, has a zero average within the cross section, and generally cannot be detected by the FBG. In contrast, the uniform (over the cross section) component $\varepsilon_{xx}^{nl}$ (associated with the integral term in Equation (2)) is used to measure the fiber’s vibration frequency with the FBG. Since this strain is dictated by the beam’s deflection, the minimal amplitude of the fiber’s vibrations should be found to ensure reliable frequency detection by FBG. In this context, larger amplitudes are preferable from the strain detection point of view. On the other hand, the vibrational amplitudes should be sufficiently small, compared to the fiber’s diameter, to avoid nonlinear effects and ensure linear frequency response. The second aspect is the actuation force required to achieve the required amplitude. Both these tasks are addressed using the reduced-order (RO) model of the beam.

In the framework of the Galerkin method [37], the deflection of a double-clamped beam loaded by a uniformly distributed force $p_{0}$ is approximated by the expression

(8) $$w\left(x,t\right)\approx w_{m}\left(t\right)\;\varphi \left(x\right)\;\;$$

where φ(x)=0.5[1−cos(2πx/L)] is the fundamental buckling mode of the beam used here as the base function. By substituting Equation (8) into Equation (1), multiplying by φ(x) and integrating over the length of the beam, we obtain the ordinary differential equation of the beam with a circular cross section, which can be written in the form

(9) $$\frac{1}{{\tilde{\omega }}_{0}^{2}}{\ddot{w}}_{m}+\frac{2\zeta }{{\tilde{\omega }}_{0}}{\dot{w}}_{m}+\left[1+\frac{N_{0}}{N_{E}}+\frac{w_{m}^{2}}{d^{2}}\right]w_{m}=\frac{p_{0}L^{4}}{4\pi^{4}EI}$$

where ${\tilde{\omega }}_{0}=4\pi^{2}\sqrt{EI/(3\rho AL^{4})}$ is the approximation of the fundamental mode circular frequency, $2\zeta =cL^{2}\sqrt{3/(16\pi^{4}EI\rho A)}$ is the equivalent damping factor and the overdot $\dot{\left(\cdot \right)}$ denotes time derivative. Note that since the bucking rather than vibrational mode was used as the base function, the approximate eigenvalue $2\pi /\sqrt[{4}]{3}\approx 4.77$ differs from the exact value of $\lambda_{1}\approx 4.73$.

By substituting Equation (8) into the static counterpart of Equation (2), the expression for the nonlinear, motion-induced component of the strain $\varepsilon_{xx}^{nl}$ can be obtained

(10) $$\varepsilon_{xx}^{nl}\approx \frac{\pi^{2}\;w_{m}^{2}}{4L^{2}}$$

Note that under bending, the resulting axial, cross-section-averaged strain in the fiber is positive, regardless of the deflection direction, and proportional to the square of the deflection amplitude. The dependence of the motion-induced strain on the fiber vibrational amplitude is shown in Figure 2b for different beam lengths. In accordance with Figure 2b, the excitation of the fiber’s vibrations with amplitudes of several micrometers (much smaller than the fiber’s diameter) is sufficient to induce axial strain above a few με noise levels of commercial equipment operated in static measurement mode.

A few words should be said about the sensor’s possible measurement range, which could be limited for several reasons. The tensile strength of optical fibers might vary in a wide range, between 400 MPa and nearly 5 GPa, depending on the material composition, manufacturing techniques, and presence of the protective coating or jacket. The strength is also influenced by the grating inscription method. The corresponding ultimate strain is within the range between 0.5% (5 mε) and 7% (70 mε) [39,40]. Based on practical experience, a conservative design limit of 700 MPa, corresponding to approximately 10 mε, is commonly adopted. In the case of compression, the critical Euler’s buckling strain $\varepsilon_{xx}^{E}=N_{E}/\left(EA\right)=\left(\pi^{2}/4\right){\left(d/L\right)}^{2}$ for slender beams is typically much smaller than the ultimate strength value. For the 125 μm diameter fiber, the buckling strain varies between 250 με and 1500 με for fiber lengths of 12 mm and 5 mm, respectively. Note, however, that in resonant sensors the measurement range is mainly limited by the nonlinearity of the SF rather than by the strength or buckling [24].

The actuating force $p_{0}$ required to reach the static midpoint displacement $w_{m}=\left(2L\sqrt{\varepsilon_{xx}^{nl}}\right)/\pi$ can be obtained by solving the static counterpart of Equation (9) where the necessary motion-induced strain $\varepsilon_{xx}^{nl}$ is found using Equation (10). Our calculations show that for the adopted dimensions the effective stiffness of the fiber is dominated by the bending stiffness and deflection-induced stretching rather than by the pre-stress $N_{0}$. The resultant actuating force $P_{0}=p_{0}L$ required to achieve the strain $\varepsilon_{xx}^{nl}$ in the case of $N_{0}=0$ and under static operation is shown in Figure 2c,d for the 70 μm and 125 μm diameter fibers, respectively. The strain of $\varepsilon_{xx}^{nl}\approx$ 100 με can be achieved by application of the actuating force of $p_{0}=4.75$ N/m for an 8 mm long fiber and $p_{0}=1.65$ N/m for a 12 mm long fiber. Since the beam is operated at resonance, the vibrational amplitude is estimated by multiplying the static deflection by the quality factor *Q*. As a result, the value of the required actuating force obtained using the static approach should be divided by *Q*. In our experiments in air, the value of Q≈ 70 was measured. In the case of operation in vacuum, the typical quality factors of the beams of similar dimensions made of quartz may vary between several thousand and up to several tens of thousands [41].

Here we evaluate the necessary actuating force for three possible actuating scenarios. The first is inertial actuation, which was actually used in this work and achieved by mounting the fiber on a vibrating shaker. In this case, the distributed force is $p_{0}=\rho A\omega^{2}w_{a}$, where $w_{a}$ is the actuation amplitude of the shaker and ω is actuation frequency. For 8 mm long fiber actuated close to resonance at f≈ 10,000 Hz (see Figure 2a), we obtain that to reach the required force per unit length of $p_{0}^{\mathrm{req}}=4.75$ N/m (or the resultant force $P_{0}=p_{0}L=38$ mN), the required shaker amplitude is $w_{a}^{\mathrm{req}}=11.1/Q\;\mu$m, Figure 2c,d. This means that even for a low-Q actuation scenario, inertial vibration excitation is feasible. Another possible scenario is a magnetic actuation by a Lorentz force such that $p_{0}=Bi$ where *B* is the magnetic flux density and *i* is the electric current. In this case, for *B* in the 1 T range, low Q excitation may require an electric current of a few hundred mA, while for $Q\approx 10^{4}$, sub-mA current might be sufficient.

Finally, it is instructive to estimate the electrostatic force which can be applied to a cylindrical beam using a gap-closing electrode. The capacitance of the cylinder of length *L* and diameter *d* located above an infinite plane at the distance $g_{0}$ is given by the expression

(11) $$C=2\pi \varepsilon_{0}\ln^{-1}\left[\frac{g_{0}}{d/2}+\sqrt{{\left(\frac{g_{0}}{d/2}\right)}^{2}-1}\right]$$

where $g_{0}$ is the perpendicular distance from the center of the cylinder to the plane. The force is calculated by differentiating the capacitor energy $W=CV^{2}/2$ with respect to the gap. For $g_{0}-d/2=20\;\mu$m and V=100 V, we obtain the quasi-static value of $p_{0}\approx 8$ mN/m. While static actuation in this case requires a high actuating voltage, actuation in vacuum at Q of the order of several thousand is feasible. Our calculations therefore show that standard optical fibers with a length of about several mm are suitable for use as the vibrating elements. Furthermore, by sealing the system in vacuum and ensuring low-loss fiber connections, the Q-factor can be larger by several orders of magnitude. This opens up the possibility of actuating the system using weak forces inherent in the system, such as noise or structural vibrations.

## 3. Experiment

### 3.1. Experimental Setup

An experimental setup was built in order to conduct several types of experiments, each pursuing different goals. The goals were: (i) to demonstrate that the optical fiber can serve as a mechanical vibrating element; (ii) that the FBG sensor embedded in the fiber can be used for interrogating the vibrating fiber’s resonant response, and (iii) to demonstrate the dependence between the fiber’s tension and the resonant frequency measured using FBG.

In all the experiments, a standard silica (SiO_2_) fiber of 125μm in diameter was used. A grating, about 1–2 mm in length, was inscribed into the 9μm silica core of the fiber. The reflection band of the fiber was chosen to be within the spectral range of the measurement equipment (1510–1640 nm). The fiber was coated with a 12.5 μm thick polyimide coating, making the total diameter 150μm. Prior to conducting the experiments, this coating was removed near the FBG location to enable the writing of the grating into the fiber’s core. This section was then re-coated with polyimide but with a less accurate thickness, and the total diameter was approximately 330 μm.

**Figure 3:**
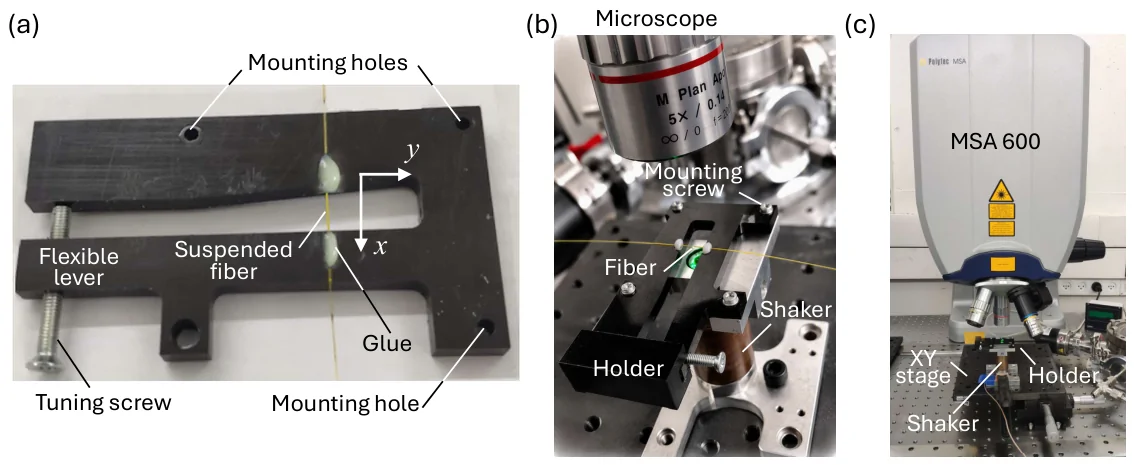
(**a**) An optical fiber attached to the fiber holder. The axial strain of the fiber is adjusted by turning the tuning screw. (**b**) The holder attached to a shaker under a microscope. (**c**) The holder with the fiber and the shaker located under the scanning laser Doppler Vibrometer MSA 600.

A key component of the experimental setup is a custom-built Delrin-made CNC-manufactured fiber holder (a loading frame) used to clamp the ends of the suspended segment of the fiber and to provide an ability to apply and adjust the axial stretching of the fiber, Figure 3a. The U-shaped holder incorporates two flexible levers, four mounting holes, and a tuning screw. The footprint of the holder was 70 mm × 38 mm × 4 mm. The fiber is attached, using glue (Epoxy-DP460NS), to the top surface of the holder. The fiber segment with two fixed ends forms a double-clamped beam configuration. Extra attention was given to keep the suspended fiber segment straight by positioning the fiber in two V-grooves engraved into the top surface of the holder. The length of the hung fiber at this location is approximately 8 mm. The holder contains two flexible levers connected by a tuning screw. By turning the screw, the distance between the fixed ends of the suspended fiber segment, and therefore the axial stretching of the fiber, can be changed in a controllable manner. The holder is mounted, using screws, on a vertically moving piezoelectric shaker allowing excitation of the vibrations of the fiber in the vertical (*z*) direction and positioned under a 5× objective of an optical microscope, Figure 3b.

The dynamic axial strain experienced by the vibrating fiber was registered using several approaches. First, a standard FBG interrogator (MicronOptics sm130, sampling rate ≤1 kHz) was used for static measurement of the fiber’s axial strain controlled by the holder’s screw. This interrogator determines the wavelength of peak reflection of the FBG following a time-consuming scan of its (optical) spectrum of operation (80 nm). Consequently, it is not suitable for measuring the resonant response of a fiber vibrating at mechanical frequencies in the tens of kHz range. Therefore, a much faster technique was exploited comprising a tunable laser, and a fast optical power sensor, whose output voltage was digitally analyzed by an electrical spectrum analyzer [42,43]. In this technique, the laser wavelength is set on the mid-reflection wavelength of the FBG spectrum at rest mode. When the fiber is subjected to vibration-induced strain, the reflected power at this wavelength changes in time due to the resulting shift in the Bragg spectrum. This time-varying intensity signal is captured by the optical sensor and its corresponding Fourier transform can be analyzed for the extraction of the mechanical motion.

**Figure 4:**
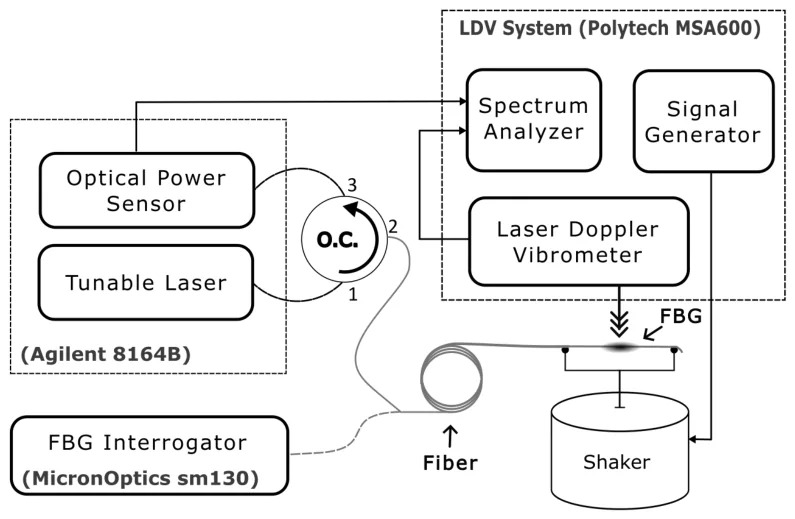
Functional diagram of the experimental setup (o.c.—optical circulator: Light enters the circulator at port 1, coming out at port 2, heading towards the FBG. The return from the FBG enters the circulator at port 2, and finally emerging at port 3). Signal generator and spectrum analyzer are built-in features of the MSA600 signal processing module.

For in situ verification of the FBG readout, the fiber oscillations were registered using laser Doppler vibrometry (LDV). Specifically, the Polytech MSA600 Micro System Analyzer was used. To focus the interrogating laser spot on the central point of the suspended segment of the fiber, the shaker with the fiber holder was mounted on a micrometric xy stage, while the vertical *z*-direction alignment was adjusted by moving the MSA head, Figure 3c. In all experiments, the out-of-plane single-point velocity mode of the LDV was used. The acquired time-domain data were then FFT-processed to obtain the resulting spectral response. The functional diagram of the experimental setup is depicted in Figure 4.

### 3.2. Experimental Procedure

First, to obtain the baseline strain values corresponding to the initial configuration of the fiber, we measured the optical reflectance spectrum using the sm130 FBG interrogator immediately after gluing the fiber to the frame and mounting the assembly on the shaker. In this baseline experiment, the LDV was switched ON to account for the influence of the thermal strain induced by the laser heating. Next, we measured the mechanical resonant response and used the corresponding resonant frequency as the reference baseline value for the fiber in its initial state. Note that in our experiments, we used the LDV, an established and widely implemented technique for experimental dynamic analysis of microstructures, only for comparison with the FBG-based measurements. The response of the actual sensor with an integrated sensing fiber will be monitored solely by the FBG, thereby eliminating LDV heating.

In all the experiments, a nominally 8 mm ± 0.5 mm-long suspended fiber segment was used. A chirp voltage signal with an amplitude of 5 V and in the range of frequencies between 5 kHz and 30 kHz, supplied by the MSA, was first applied to the shaker to roughly estimate the resonance frequency. The time-domain velocity response of the fiber in the vertical *z* direction was acquired by the LDV, and the FFT analysis was performed using the MSA software.

**Figure 5:**
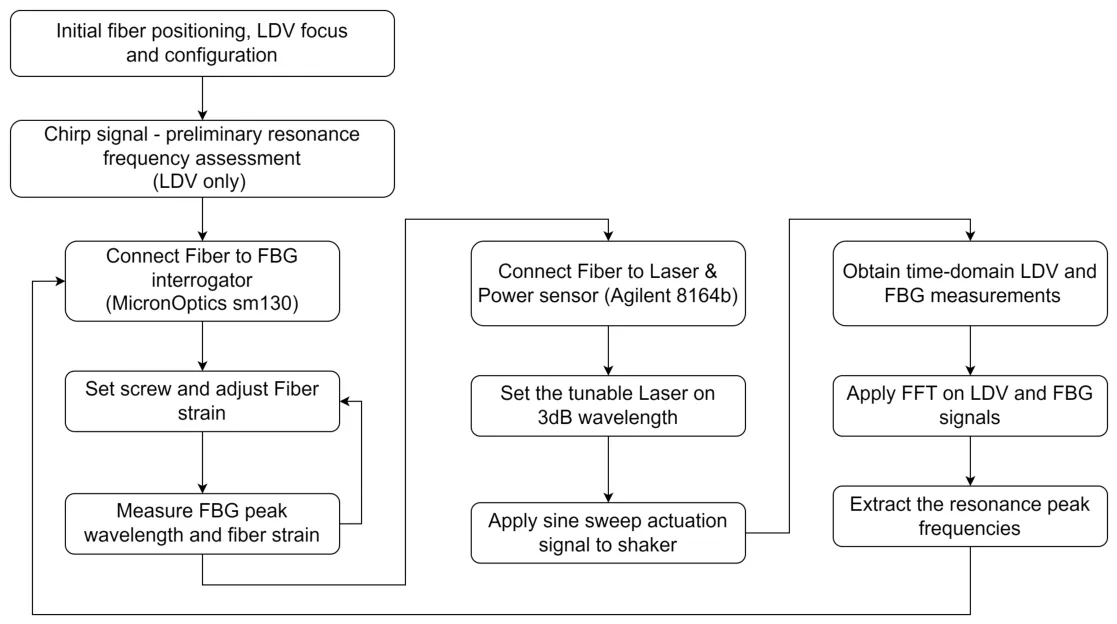
Experimental procedure flow.

The experimental workflow is detailed in Figure 5. All the measurements performed using FBG were carried out when the LDV was ON, and the effect of laser heating on the fiber’s strain and natural frequency was therefore accounted for. The following steps were repeatedly performed for different fiber axial strains:

- First, the fiber strain was set by turning the tuning screw, Figure 3. The strain readout and the reflection peak wavelength were captured with the sm130 FBG interrogator.
- Next, the fiber was disconnected from the sm130 and connected by an optical circulator to both the laser source (81640A) and the power sensor (81636B) of the fast FBG interrogation setup described above. The laser wavelength was set on the mid-reflection wavelength (“3dB wavelength”).
- Once the optical measurement was set up and the LDV interrogating beam was focused and configured, a harmonic voltage signal with an amplitude of 5 V was applied. The frequency up-sweep frequency range was set to between 7 kHz and 11 kHz.
- For every measurement, three time-domain signals were acquired: the input signal, the LDV output, and the optical FBG sensor output.
- Lastly, the corresponding frequency-domain outputs were obtained by performing an FFT of the time-history response acquired by both LDV and FBG using the PSV software (a part of the MSA). The resonant frequency values were extracted from both the LDV and the FBG signals.

In addition, at the end of each experiment sequence, while the fiber was still stretched at its final state, a static deflection test was carried out. A small metal needle connected to a micro-positioner stage was used to gently pull the fiber up and down (in the positive and negative vertical *z*-direction) with a known and controlled magnitude. The strain in the fiber was measured with the sm130 FBG interrogator. The purpose of this experiment was to verify that the fiber is straight and not initially curved or buckled, and therefore is subjected to the same additional (compared to its initial rectilinear configuration) tensile strain and consequently the same FBG output at the maximal (upward) and the minimal (downward) deflection. Note that for this reason, in the absence of imperfections, the vibration-induced additional tensile strain exhibits two peaks during one period of vibrations. For this reason, the frequency of the strain response registered by FBG is expected to be twice the frequency of the fiber vibration measured by the LDV.

## 4. Results

**Figure 6:**
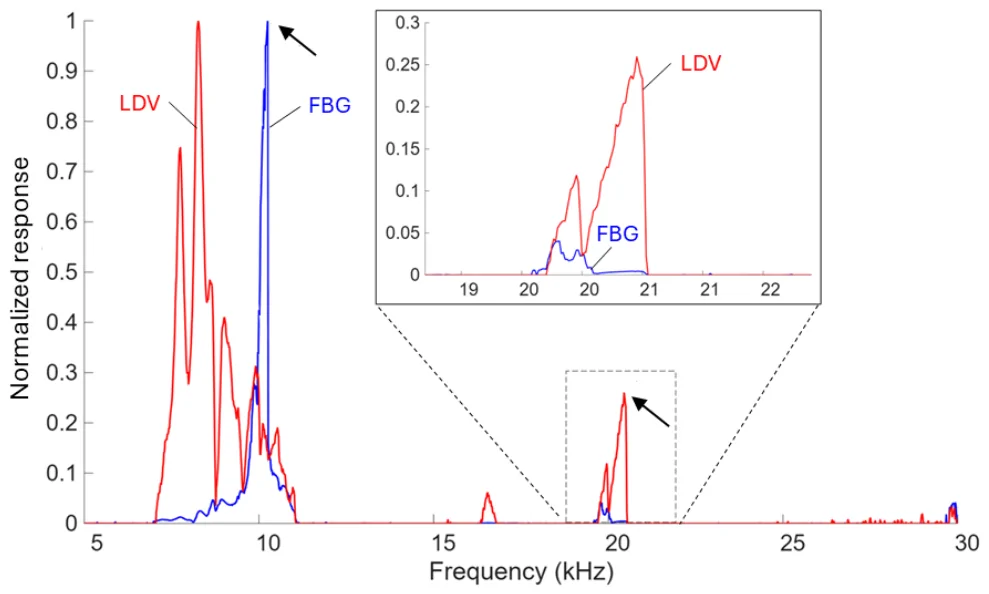
The fiber’s mechanical response in the frequency domain under static strain of 271 με as measured with the FBG and LDV. The arrows point to the dominant peaks in each of the responses. The FBG dominant peak at 20.5 kHz (zoomed-in the inset) is a double-frequency response of the same resonant movement detected by the LDV at 10.25 kHz.

Frequency responses of the fiber subjected to the axial strain of ≈271 με measured by the FBG and the LDV are shown in Figure 6. The non-Lorentzian shape of the frequency curves is an indication of the presence of nonlinearity. Note that in the weakly nonlinear system, the frequency corresponding to the peak amplitude generally differs from the resonant frequency of the associated linear system. The detailed analysis of the nonlinear response is out of the scope of this investigation and reserved for our future work. Hereafter, for the sake of simplicity, the values corresponding to the peaks of the frequency responses are used as resonant frequency indicators and referred to as “resonant frequencies”.

Comparison of the responses measured by the LDV and the FBG shows that the LDV signal contains one dominant peak at the frequency of ≈10.25 kHz. In contrast, the FBG-generated spectrum contains several peaks within the range of the frequency sweep, with an additional dominant peak in the vicinity of the frequency twice the peak frequency measured by the LDV. This behavior is attributed to the fact that the vibration-induced stretching of the beam measured by the FBG reaches a maximum twice per period. Hence, hereafter, when processing the FBG signals, we selected this peak as the one associated with the resonance at half this peak’s frequency.

**Figure 7:**
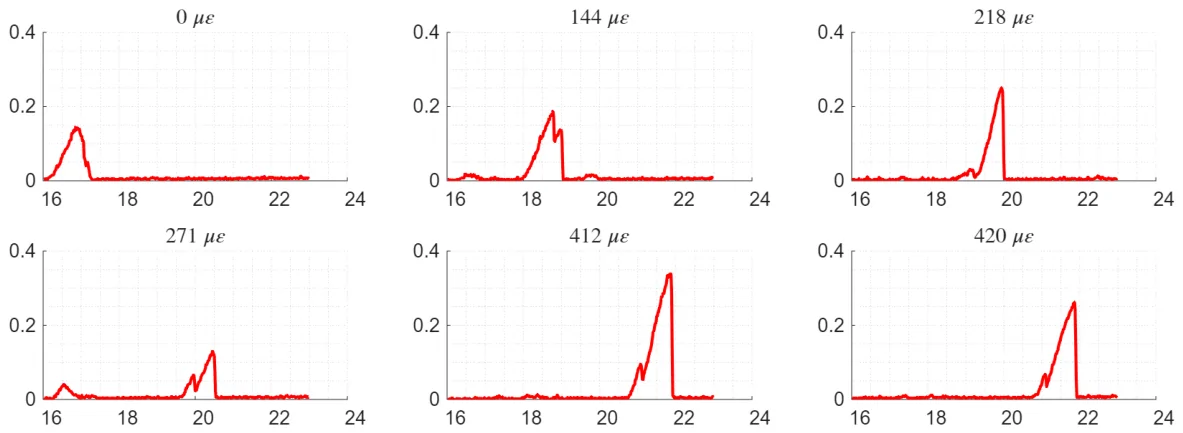
Frequency responses of the fiber measured by FBG for different strain states. X-axis: frequency (kHz), Y-axis: FBG response amplitude (V).

**Figure 8:**
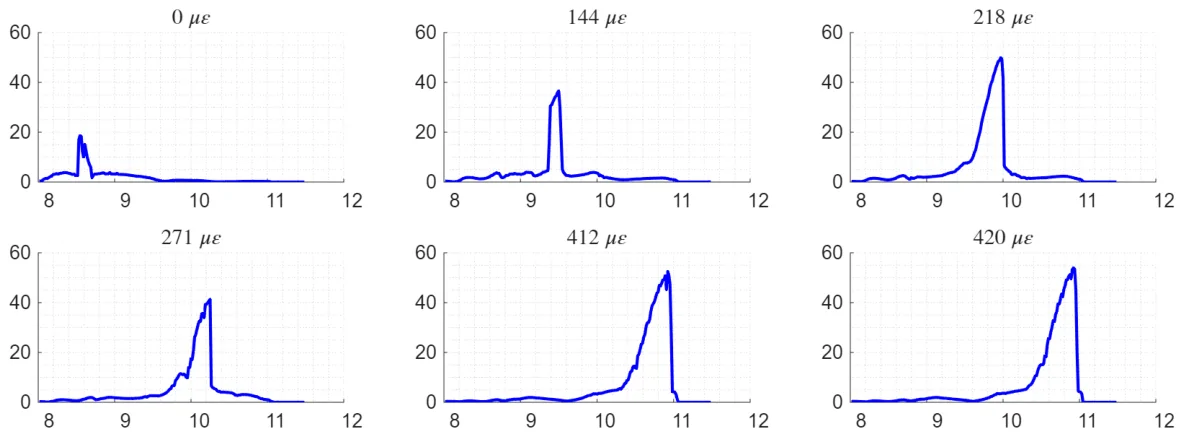
Frequency responses of the fiber measured by LDV for different strain states. X-axis: Frequency (kHz), Y-axis: vibrational amplitude (μm).

**Figure 9:**
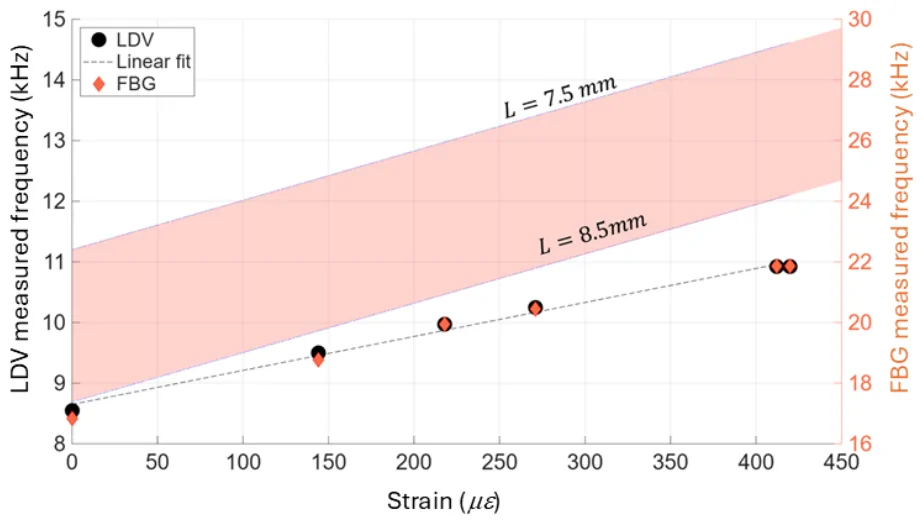
Resonant frequencies of d=125μm diameter fiber measured using LDV (black circles) and FBG (orange diamonds). The black dashed line depicts a linear fit. The colored area depicts the theoretical uncertainty range of frequencies for 8±0.5 mm-long fiber.

The response spectra registered by FBG and LDV and corresponding to six different values of the strain are shown in Figure 7 and Figure 8, respectively. A summary of the results is presented in Table 2 and in Figure 9. Note that the actual value of the strain in the fiber in the initial configuration is not known and generally can be non-zero. Thus, in Figure 7 and Figure 8, the strain corresponding to the configuration with the lowest associated mechanical frequency $f_{0}$ measured in each experiment before the application of the stretching force by a loading frame was used as the baseline value and referred to as the zero strain.

In these experiments, the range of the axial strain increase was ≈420 με, and the corresponding resonance frequency shift (as measured by the LDV) between the lowest and the highest strain states was as much as ≈2.36 kHz. The ratio between the resonant curve peaks measured by the LDV and the FBG peaks was very close to 2 in all the measurements, as expected. The relative frequency shift, $\Delta f/f_{0}$, was calculated separately for the cases of the LDV and the FBG measurements. For each measurement method, the baseline frequency $f_{0}$ was taken as the lowest frequency peak measured by this specific method. The relative scale-factor was calculated by dividing the relative frequency shift by the FBG measured strain for every measurement. The slight decrease in the sensitivity at increased strain values is attributed to the influence of nonlinearity.

Our results show that the dependence between the strain and the resonant frequency is close to linear (the linear fit residual is $R^{2}=0.99$), Figure 9. The average SF is df/dε≈ 5.6 Hz/με (which, for the adopted value of the Young’s modulus and the fiber diameter of 125 μm, is equivalent to the stress and force sensitivities of ≈80 Hz/MPa and ≈6.52 Hz/mN, respectively). The corresponding relative SF is $\Delta f/(f_{0}\varepsilon )\approx$ 682 ppm/με. Note that the strain $\varepsilon_{xx}$ was measured by the FBG before starting and after completion of the driving frequency sweep. In most cases, the strain values measured after the completion of the sweep cycle were higher than the values before the sweep. The observed hysteresis is attributed to the non-idealities of the beam’s end clamping.

Our results show that the resonant frequencies measured by the LDV and by the FBG approaches are in good agreement. However, the frequencies, Equation (4), and sensitivities, Equations (6) and (7), predicted by the model are higher than the measured values. The discrepancy between the theoretical and experimental results is attributed to high uncertainty in the fiber clamping conditions, the influence of the parasitic compliances of the glue and the frame on which the fiber was mounted, uncertainty in the fiber’s length, and possible misalignment between the fiber clamped ends, rather than to model limitations. While nonlinearity of the response has certain influence, Figure 6, Figure 7 and Figure 8 indicate that the difference between the natural frequencies of the linearized problem and the peak values of the frequency responses used as the frequency indicator is less than 10% in all cases. To estimate the influence of the uncertainty in the fiber’s length, the frequencies were calculated for a realistic range of the beam length between 7.5 mm and 8.5 mm. The results shown in Figure 9 indicate that while the calculated frequencies are higher than the experimental values, as expected, the sensitivity (the slope of the strain–frequency curve) is not affected by the beam’s length uncertainty, Equation (6). Note that despite its approximate character, the model illustrates qualitatively and also quantitatively the feasibility of the proposed operational principle of the sensor.

## 5. Implementation in a Vibrating Beam Accelerometer

In this work, as an example of the suggested FBG-based resonant strain sensing application, we investigate the applicability of the approach for possible implementation in high-end vibration beam accelerometers. Inertial sensors—micro gyroscopes and accelerometers—are probably the most intensively researched devices in the realm of resonant MEMS (see [24,41,44,45,46,47,48,49,50,51] among many others). Specifically, microelectromechanical (MEMS) accelerometers were among the first successfully commercialized MEMS devices and are nowadays widely used in various applications, starting from consumer electronics and up to high-end navigation, control, and guidance systems, due to their small size, low power consumption, cost-effectiveness, and integrability with electronics [24,46,48]. Among the promising approaches allowing performance enhancement, frequency modulation (FM)-based acceleration sensing is exploited. In vibration beam accelerometers (VBA), the natural frequency of a sensing beam attached to a proof mass (PM) is influenced by an axial force transferred to the beam by the mass [45]. Since the force transferred to the sensing beams by the PM and, consequently, the SF, are small, various force amplification mechanisms were suggested [41,48,49,52]. Resonant accelerometers demonstrate higher sensitivity, wider dynamic range, better robustness, lower Flicker (1/f) noise, and less pronounced susceptibility to external shock and vibrations when compared to their statically operated counterparts [48]. Dynamically operated accelerometers with outstanding performance compatible with navigation grade [41,51], or applicable in gravimetric applications [50,51] were developed.

Significant efforts were also devoted to the incorporation of optical sensing in micro accelerometers [53,54,55]. FBG sensing is considered one of the promising technologies in this area, due to the high precision and sensitivity of the devices, their immunity to electromagnetic interference, and their ability to provide multi-point sensing in challenging environments. These characteristics make them suitable for a wide range of applications such as structural health monitoring, seismic detection, and aerospace engineering [35,56]. Numerous designs of FBG-based, statically operated accelerometers were reported to achieve sensitivities as high as 1721.6 pm/g, wide bandwidths, and a dynamic range of up to 80 dB [53,57]. In these devices, to convert the acceleration into measurable strain, the fibers with inscribed FBG are commonly attached to host structures such as cantilevers, diaphragms, or hinges [57,58,59,60]. A few works reported on accelerometers with free-standing, suspended optical fibers, which are not attached to a host structure [61,62,63,64]. The frequency measurement of microscale free-standing cantilever-type optical waveguides fabricated using a custom-developed process was reported in [61]. An accelerometer with paralleled string-type fibers attached to a vibrating mass was reported in [62,64]. Deflection monitoring in a suspended, laterally loaded (by an inertial force) fiber used for structural vibration monitoring was reported in [63]. In all these works, to measure acceleration, the suspended fibers were operated in a quasi-static rather than resonant scenario.

Here we present the results of a model-based feasibility study of a VBA incorporating optical fiber as the sensing element and relying on FBG for the vibrating fiber frequency detection. Two architectures are considered: without (Design 1) and with (Design 2) a compliant force amplification mechanism. In both cases, the device is assumed to be fabricated from single-crystal Si using a common and well-established process based on deep reactive ion etching (DRIE). It should be noted that detailed design of VBA is a complex multistage process, involving several parametric studies and optimization cycles, which are usually necessary in order to meet target requirements and figures of merit imposed by a specific application [65]. Since the goal of the present work is to provide the basic feasibility demonstration of an FBG-based VBA, only a part of the device performance metrics are discussed. These include the sensing beam natural frequency, absolute and relative scale factors, the lowest frequency of the PM, and the overall footprint.

**Figure 10:**
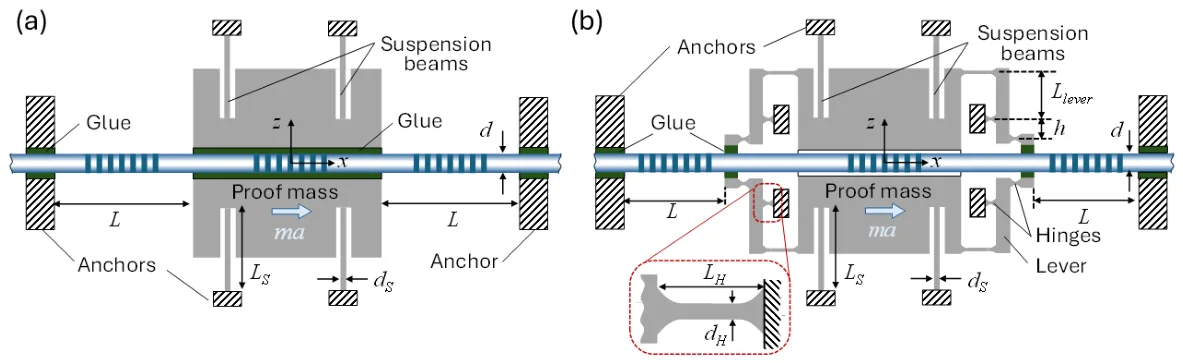
Resonant accelerometer architectures: (**a**) Design 1 without a force amplification and (**b**) Design 2 with force amplification. The parameters of the devices are detailed in Table 3.

The device shown in Figure 10a (Design 1) contains a proof mass *m* of the size $L_{m}\times L_{m}\times b$ attached to the holder by four clamped-guided suspension beams of the length $L_{S}$ and width $d_{S}$. It is assumed that the mass and the suspension beams are fabricated from a single Si wafer of thickness b=500μm. Optical fiber is integrated in such a way that each of the two suspended fiber segments with the FBG sensors is attached to the mass (for example, by glue) at one end and to an immovable anchor at the other end. Note that the part of the fiber attached to the PM moves as a rigid body together with the PM and is therefore not stretched by the inertial force. An FBG inscribed into this segment can be used, for example, for calibration of the sensor and compensation of the Temperature Coefficient of Frequency (TCF) of the sensor. Two fiber diameters are considered: d=125μm and d=70μm. In each case, the length of the fibers is chosen to provide a natural frequency in the vicinity of 50 kHz. In accordance with the results presented in Section 2, shorter beams may require higher actuating forces, while lower natural frequencies of longer beams may result in higher relative SF and higher SF nonlinearity and noise. Parameters of the device are detailed in Table 3. Note that the width $d_{S}$ of the suspension beams is chosen such that the (conservative) aspect ratio d/H≈1:10 is consistent with the etching design rules of most common fabrication processes.

The displacement of the mass in the sensing *x*-direction induced by an inertial force ma, where *a* is the acceleration, results in the tension of the left segment of the fiber and compression of the right segment. The differential frequency shift between the left and the right sensing beams, and the differential absolute and relative scale factors are defined (see Equations (6) and (7)):

(12) $$\begin{array}{ccc} \Delta f & = & f_{L}-f_{R}=f_{0}\sqrt{1+\frac{N_{a}}{N_{E}}}-f_{0}\sqrt{1-\frac{N_{a}}{N_{E}}}\approx f_{0}\frac{N_{a}}{N_{E}},\;\left(\mathrm{Hz}\right) \end{array}$$

(13) $$\begin{array}{ccc} SF^{a} & = & \frac{f_{0}g}{a}\left(\frac{N_{a}}{N_{E}}\right),\;\left(\frac{\mathrm{Hz}}{g}\right) \end{array}$$

(14) $$\begin{array}{ccc} \;SF_{\mathrm{REL}}^{a} & = & \frac{g}{a}\left(\frac{N_{a}}{N_{E}}\right)\times 10^{6},\;\left(\frac{\mathrm{ppm}}{g}\right) \end{array}$$

where $N_{a}=EAu/L$ is the axial force acting on the fiber segment, $u=u_{m}$ is the elongation of the fiber segment, and $u_{m}$ is the proof mass displacement in the *x* direction. Note that Equations (13) and (14) are multiplied by the acceleration of gravity g=9.81 m/s^2^ to give, in common, the SF in Hz/g. The details of the calculation of the force $N_{a}$ are presented in the Appendices Appendix A and Appendix B.

**Figure 11:**
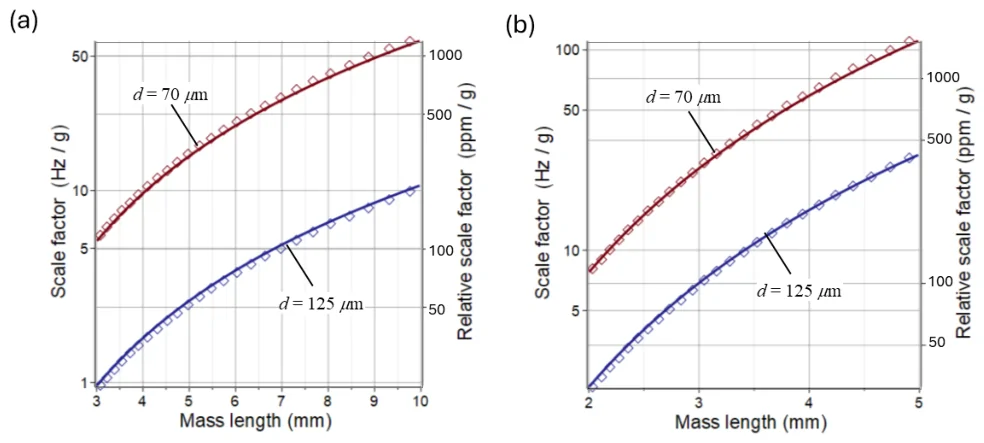
Differential scale factor $SF^{a}$ and relative scale factor $SF_{\mathrm{REL}}^{a}$ of the resonant accelerometers without (**a**) and with (**b**) force amplification. The parameters of the devices are detailed in Table 3. Solid lines and markers correspond to the absolute and relative scale factors, respectively.

The absolute and the relative sensitivities of the device for differing sizes of the PM are shown in Figure 11a. Our results suggest that even without force amplification, the absolute differential SF of the sensor is in the range between a few Hz/g and up to 10 Hz/g for the 125 μm diameter fiber and may exceed 50 Hz/g in the case of the 70 μm diameter fiber. These values can be increased in principle by enlarging the PM; however, at the expense of a lower fundamental mode frequency of the device (which, in the considered case, is the out-of-plane, *z*-direction vibration of the mass). Our calculations show that for a 10 mm × 10 mm × 500 μm mass, the lowest frequency is ≈4 kHz, which is within a generally acceptable range. Note that the sensing beam geometries, natural frequencies, and obtained SF values are generally consistent with the figures of merit of high-end VBAs previously reported in the literature [41].

The device shown in Figure 10b (Design 2) incorporates a force amplification compliant mechanism. While numerous architectures of these mechanisms were reported [48,51], we implement here one of the earliest and most common architectures [38]. In this device, the moving end of the suspended fiber segment is not attached directly to the PM but is attached to the force-amplifying levers. The levers, each of length $L_{lever}=L_{m}/2$ are attached to the substrate and to the fiber’s end using flexible (pseudo-) hinges, as shown in the inset of Figure 11b. Due to the offset $h\ll L_{lever}$ between the hinges, the displacement of the mass results in the rotation of the levers and amplification of the force stretching or compressing the sensing beam. Note that since the displacements *u* of the moving ends of the fibers differ from the displacement $u_{m}$ of the PM, the fiber is not attached to the PM itself, and only the sensing fiber’s segment ends are glued to the compliant mechanism. The device is assumed to be fabricated from the b=100μm thick device layer of the silicon-on-insulator (SOI) wafer. The geometric parameters of the device, Table 3, are compliant with the common fabrication process limitations.

The results shown in Figure 11b indicate that the implementation of the force amplification mechanism allows an increase in the SF and a decrease in the PM size and therefore of the overall footprint of the devices. Namely, for a 5 mm × 5 mm × 100 μm mass, the differential SF of more than 100 Hz/g can be reached while preserving the lowest mass frequency to be higher than 7 kHz. Note that while further increase of the SF can be achieved in principle in this device, higher values of the relative scale factor may result in higher SF nonlinearity. We leave this analysis for future work.

## 6. Conclusions

In this work, a new approach for optical strain or force sensing using a fiber Bragg grating is introduced and experimentally demonstrated. The key innovative feature of the proposed concept is that the suspended optical fiber serves as a resonant beam-type sensing element, whose natural frequency is influenced by the fiber’s axial strain. In the proposed sensing scenario, the vibrational response of the stretched or compressed beam is monitored via FBG interrogation, and the resonant frequency is extracted. The feasibility study carried out using a reduced-order model of the suspended fiber-beam shows that beams of realistic dimensions, compatible with commercially available off-the-shelf fibers with diameters of 125 and 70 μm, can be used for highly accurate strain sensing. The model results are consistent with the experimental findings obtained using a custom-built loading and measurement setup. Finally, the applicability of the proposed concept for implementation in a vibration beam accelerometer is demonstrated using the model. The proposed approach has several beneficial features. Our model and experimental results collectively show that the relative sensitivity of the frequency-based measurement can be up to two orders of magnitude higher than that of direct, commonly adopted static FBG-based strain sensing. This feature enables detection of extremely small strains or forces below the usual FBG sensor limits. Moreover, high-frequency operation may improve the signal-to-noise ratio and reduce flicker noise. One of the key, and probably most beneficial, features of FBG detectors is multisensing, namely the ability to measure strain at multiple locations along the fiber. The proposed approach extends this ability from purely spatial multiplexing to frequency-domain-based signal-source localization. Several signal processing algorithms can be exploited to leverage this feature and can be a topic for future research.

The proposed strain/force sensing concept can be efficiently used in a diversity of resonant sensors, including but not limited to pressure, acoustic, magnetic, and electric field sensors, mass, gas, and biosensors. One of the promising applications explored in the present work is a vibration beam accelerometer (VBA). Our model results show that by incorporating off-the-shelf optical fibers into devices with realistic, manufacturable PM and structural element dimensions, performance comparable to reported state-of-the-art VBAs can be achieved. However, the key beneficial feature of the proposed approach is its multisensing capability, which is unique to the FBG-interrogated sensors. This ability may open new interesting opportunities for data-driven applications of MEMS accelerometers for industrial and environmental condition monitoring [66] or reservoir computing [67].

## Figures and Tables

**Table 1:** Properties of a standard optical fiber.

Parameter	Symbol	Value
Material	SiO_2_	(glass)
Young’s modulus	*E*	70 GPa
Density	ρ	2200kg/m
Diameter	*d*	125μm
Cross-section area	*A*	$\pi d^{2}/4$
Second moment of area	*I*	$\pi d^{4}/64$

**Table 2:** Experimental results.

$\varepsilon_{0}$	$\varepsilon_{1}$	f_LDV_	Q_LDV_	f_FBG_	Q_FBG_	f_FBG_	Δf_LDV_	Δf_FBG_	SF_LDV_	SF_FBG_
(με)	(με)	(kHz)		(kHz)		/f_LDV_	(rel.)	(rel.)		
0	15	8.55	61	16.86	32	1.97	0	0		
144	171	9.50	61	18.77	48	1.98	0.112	0.114	712	726
218	248	9.97	34	19.94	67	2.00	0.166	0.184	713	784
271	328	10.25	44	20.50	73	2.00	0.199	0.214	635	690
412	443	10.92	29	21.86	61	2.00	0.278	0.298	648	693
420	448	10.91	30	21.84	64	2.00	0.278	0.298	637	682

ε_0_—strainbefore vibrations. ε_1_—strain after vibrations. f_LDV_, f_FBG_—resonance peak in LDV/FBG response. Q_LDV_, Q_FBG_—Q factor of the resonance peak in the LDV/FBG response. f_FBG_/f_LDV_—ratio between the resonance peaks in FBG and LDV responses. Δf_LDV_, Δf_FBG_—relative frequency-shift ($\Delta f/f_{0}$) from LDV/FBG measurement. SF_LDV_, SF_FBG_—relative scale factor (ppm/με) from LDV/FBG measurement.

**Table 3:** Parameters of the devices used in calculations. Values without and with parentheses correspond to the optical fiber diameters of d=125μm (d=70μm).

Parameter	Design 1		Design 2	
	Value	Unit	Value	Unit
Modulus of elasticity of Si $E_{Si}$	169	GPa	169	GPa
Mass density of Si $\rho_{Si}$	2300	kg/m^3^	2300	kg/m^3^
Sensing beam length *L*	3.5 (2.7)	μm	3 (2.25)	mm
Suspension length $L_{S}$	3	mm	2	mm
Suspension width $d_{S}$	50	μm	10	μm
Thickness *b*	500	μm	100	μm
Hinge length $L_{H}$	-	-	50	μm
Hinge width $d_{H}$	-	-	10	μm
Lever length $L_{S}$	-	-	$L_{m}/2$	-
Offset *h*	-	-	25 (35)	μm
Sensing beam frequency $f_{0}$	51.238 (48.215)	kHz	69.741 (69.431)	Hz

## Data Availability

The raw data supporting the conclusions of this article will be made available by the authors on request.

## Abbreviations

The following abbreviations are used in this manuscript:

FBG	Fiber Bragg grating
SF	Scale factor
VBA	Vibrating beam accelerometer
PM	Proof mass

## Appendix A. Model of a Resonant Accelerometer

### Appendix A.1. Resonant Accelerometer Without Force Amplification

The equilibrium of the resonant acceleration sensing device shown in Figure 10a is described by the equation

(A1) $$(4k_{S}+2k_{t})u=ma$$

where $k_{S}=12E_{Si}I_{S}/L_{S}^{3}$ is the bending stiffness of each of the four proof mass elastic suspension beams and $k_{t}=EA/L$ is the axial (tensile) stiffness of each of the suspended optical fiber segments with the cross-sectional area $A=\pi d^{2}/4$, length *L* made of glass with the modulus of elasticity *E*. The suspension is realized as four clamped-guided beams with the length $L_{S}$, width $d_{S}$ and thickness *b* (in the *y* direction). Here, $E_{SI}$ is the elastic modulus of Si; the cross-sectional area and the second area of the suspension beams are $A_{S}=bd_{S}$ and $I_{S}=bd_{S}^{3}/12$, respectively. Since the displacement of the proof mass is small compared to the width of the suspension beams $d_{S}$, and for simplicity, we neglect the structural nonlinearity of the suspension beams.

By solving Equation (A1) in terms of *u* and substituting the outcome into Equations (12)–(14) where $N_{a}=k_{t}u$, the frequency shift Δf and the absolute $SF^{a}$ and relative scale factors $SF_{\mathrm{REl}}^{a}$ are obtained. The lowest, fundamental mode frequency of the proof mass (in the *y*-direction) is estimated using the expression

(A2) $$f_{y}=\frac{1}{2\pi }\sqrt{\frac{k_{y}}{m}}$$

where

(A3) $$k_{y}=4\times \frac{12E_{Si}d_{S}b^{3}}{12L_{S}^{3}}+2\times \frac{12EI}{L^{3}}$$

## Appendix B. Resonant Accelerometer with Force Amplification

The device shown in Figure 10b incorporates two suspended optical fiber segments with the axial tension stiffness $k_{t}$, four clamped-guided suspension beams, each with the stiffness $k_{S}$ (see Equation (A1)), and eight pseudo hinges, realized as cantilevers with the length $L_{H}$, width $d_{H}$ (see inset in Figure 10b) and with the bending stiffness $k_{H}=E_{Si}I_{H}/L_{H}$. The potential energy of the device is

(A4) $$U=8\left(\frac{1}{2}k_{H}\theta^{2}\right)+2\left(\frac{1}{2}k_{t}u^{2}\right)+4\left(\frac{1}{2}k_{S}u_{m}^{2}\right)-mau_{m}$$

Here θ is the rotation angle of the amplifying levers, *u* is the displacement of the optical fiber segment end attached to the force amplifier and $u_{m}$ is the displacement of the proof mass in the sensing *x*-direction. Since the deformation of appropriately designed beams connecting the proof mass and the levers is significantly smaller than that of the pseudo hinges, the strain energy of these beams is neglected. By using the kinematic relations (under an assumption of small rotation angles θ≪1

(A5) $$\;\theta =\frac{u}{h},\;u_{m}=\frac{uL_{lever}}{h}$$

and implementing the stationary potential energy principle, the equation of equilibrium is obtained

(A6) $$\left[8\frac{k_{H}}{h^{2}}+2k_{t}+k_{S}A_{0}^{2}\right]u=maA_{0}$$

where $A_{0}=L_{lever}/h$ is a geometrical amplification. By solving Equation (A6) in terms of *u*, the axial forces $N_{a}=k_{t}u$ acting on each of the suspended fiber’s segments can be calculated

(A7) $$N_{a}=\frac{ma}{2}A_{0}{\left[1+A_{0}^{2}k_{S}/\left(2k_{t}\right)+4k_{H}/\left(k_{t}h^{2}\right)\right]}^{-1}$$

Then using Equations (12)–(14), the relative $SF_{\mathrm{REL}}^{a}$ and the absolute $SF^{a}$ and scale factors are obtained.

Equation (A7) suggest that the actual force amplification $A=N_{a}/\left(ma\right)$ is smaller than the geometric one $A_{0}=L_{laver}/h$ and, for the adopted values of the stiffness parameters $k_{t},\;k_{S},\;k_{H}$, is determined by the offset *h*. The optimal value $h_{\mathrm{opt}}$ of the offset corresponding to the maximal amplification and therefore maximal SF is found by differentiating Equation (A7) and solving for *h* [65]

(A8) $$h_{\mathrm{opt}}=\sqrt{L_{lever}^{2}\frac{k_{S}}{2k_{t}}+4\frac{k_{H}}{k_{t}}},\;A_{\max}=A\left(h_{\mathrm{opt}}\right)=\sqrt{\frac{k_{t}}{8\left(k_{S}+8k_{H}/L_{lever}^{2}\right)}}$$

Since it was adopted that $L_{lever}=L_{m}/2$, $h_{\mathrm{opt}}$ depends on the mass size. However, this dependence is relatively minor. For the adopted constant values of $k_{t},\;k_{S},\;k_{H}$, $h_{\mathrm{opt}}$ varies between 19.8 μm and 27.2 μm for the 125 μm diameter fiber, and between 30.6 μm and 42.5 μm for the 70 μm diameter fiber.
